# Comparison of orthopaedic specialty registrar training opportunities in trusts with and without an elective surgical hub: a review of administrative data

**DOI:** 10.1308/rcsann.2024.0085

**Published:** 2025-07-11

**Authors:** EO Ojelade, WK Gray, TWR Briggs

**Affiliations:** ^1^ NHS England, UK; ^2^ Royal National Orthopaedic Hospital NHS Trust, UK; ^3^ Royal Free London NHS Foundation Trust, UK

**Keywords:** Orthopaedics, Education, Elective surgical procedures

## Abstract

**Introduction:**

During the COVID-19 pandemic in England, orthopaedic surgery trainees experienced a reduction in training opportunities due to periods of elective surgery suspension. We aimed to explore training opportunities for orthopaedic trainees in trusts with and without access to an elective surgical hub.

**Methods:**

This retrospective analysis of administrative data used eLogbook data for registrars who performed six high-volume, low-complexity orthopaedic procedures in NHS hospitals in England between April 2017 and March 2023. Data included training grade, role in the procedure, trust where the procedure was performed and procedure date. These were linked to Hospital Episodes Statistics (HES) data on the total number of these procedures conducted during the study period and whether the trust hosted or fed into an elective surgical hub at the time of the procedure.

**Results:**

Data were analysed for 1,755 trainees acting as first surgeon in 125,759 procedures. Trusts with access to an elective surgical hub significantly increased the proportion of procedures conducted by a trainee compared with non-hub trusts over the study period. Most of the increase in trainee involvement was associated with more senior trainees (ST6-8). The proportional increase was not enough to offset the decline in the absolute number of procedures conducted by trainees (25,598 (2017–2018), 21,057 (2022–2023)).

**Conclusions:**

Elective surgical hubs have made a positive contribution to training opportunities for orthopaedic trainees but not enough to offset the post-pandemic fall in activity. The number of procedures conducted in NHS hospitals and the rate of training opportunities must be increased.

## Introduction

The onset of the COVID-19 pandemic in March 2020 led to a national suspension of elective surgery in the UK.^1^ Surgery was started in phases during the second half of 2020, but partially suspended again in January 2021, with a gradual increase in activity from April 2021. This severely impacted surgical training, with elective trauma and orthopaedic surgery being the hardest hit specialty.^2^ As a result, training for many specialty trainees in years three to eight (ST3-8) was extended with special derogations implemented.^3^ This extension period came to an end on 30 September 2023.^4^

In order to address the impact of the COVID-19 pandemic on the NHS in England, the UK government and NHS England published “*Delivery plan for tackling the COVID-19 backlog of elective care*” in February 2022.^5^ The report focussed on four areas: increasing health service capacity, prioritising diagnosis and treatment, transforming the way elective care is provided and providing better information and support to patients. In 2019, the Getting It Right First Time (GIRFT) programme, which is part of NHS England, had recommended that, to transform elective care provision, existing surgical hubs should ensure that they are as productive as possible and new elective surgical hubs should be created. Elective surgical hubs perform exclusively planned surgery with ‘ring-fenced’ facilities and staff. Immediately post-pandemic, the priority for the hub sites was to conduct surgery classed as high volume low complexity (HVLC), with the aim of providing surgery for 48 weeks per year, reducing the backlog and improving outcomes for patients awaiting these common procedures.^6^ More recently, this remit has been expanded to help reduce the backlog of all patients awaiting surgical treatment. Elective surgical hubs existed prior to the COVID-19 pandemic but more are being created and accredited by the GIRFT programme. Accreditation is a mark of quality, and a commitment to training junior doctors and other members of staff is a key requirement for accredication.^7^

We aimed to compare the training opportunities available to specialty registrars in orthopaedic training programmes at NHS hospital trusts in England with and without access to (hosting or feeding into) an elective surgical hub.

## Methods

### Study design

This was a retrospective analysis of administrative data.

### Ethics

This study did not directly involve patients or NHS employees as participants and so consent was not required. The data were acquired with the approval of the Intercollegiate Surgical Curriculum Programme (ISCP) Data Analysis Audit and Research Group (DAARG) (approval number DRF0076). All data were pseudonymised at source prior to analysis. The presentation of data follows current NHS England guidance for use of administrative data for research purposes.^8^

### Timing, setting, inclusion criteria and data sources

We included data for the six elective orthopaedic procedures defined as HVLC by the GIRFT programme (anterior cruciate ligament reconstruction (ACLR), bunion surgery (BS), therapeutic shoulder arthroscopy (TSA), primary total hip replacement (THR), primary total knee replacement (TKR) and unicompartmental knee replacement (UKR)) conducted in England from 1 April 2017 to 31 March 2023.

Data were from three sources that were linked at an NHS hospital trust level:

1. The Joint Committee on Surgical Training (JCST) eLogbook database. The JCST is an advisory body to the four surgical Royal Colleges of the UK and Ireland and deals with all matters relating to postgraduate surgical training.^9^ The JCST is also responsible for the ISCP, which provides the framework for surgical training in the UK.^10^ Learning, teaching and feedback is captured using online resources, including the eLogbook, which surgical trainees use to record their operative experiences throughout their training programme.^11^ All surgical procedures in England that involve a trainee are recorded in eLogbook, including details of surgical training level (ST3-8), date of procedure, role of the trainee in the procedure (performed (P), supervised – trainer unscrubbed (S-TU), supervised – trainer scrubbed (S-TS) and assisted, where P, S-TU and S-TS mean the trainee was the first surgeon) and the NHS hospital trust where the procedure was conducted. Anonymised eLogbook data for hospitals in England were requested for the six included procedures conducted during the study period.
2. The Hospital Episodes Statistics (HES) dataset. The HES dataset contains data for all hospital admissions in England including details of procedures, diagnoses, dates of admission and discharge and patient demographics. Data were extracted from HES for the six procedures listed above during the study period using the criteria set out in Supplementary material Table S1.
3. A dataset collated by the GIRFT programme on details of all elective surgical hubs in England, including date of hub opening, date of starting orthopaedic surgery at the hub and the trusts hosting and feeding into the hub.

### Exclusion criteria

Cases were excluded where:

• the procedure was not performed in England;
• the procedure was performed at a non-NHS hospital (i.e. private/independent sector);
• the person performing the procedure was not registered with the ISCP at the time of the operation;
• the person performing the procedure had completed their registrar/specialty training when the procedure was performed, or had an incomplete training record with ISCP due to a pause in or stopping training before completion of the programme and more than 18 months had passed between the last registered grade start date on ISCP and the procedure being performed;
• the registrar/specialty trainee recorded their involvement in a procedure as assisted rather than S-TS, S-TU, P.

### Exposure of interest

The exposure of interest was a procedure performed in a NHS hospital trust hosting or feeding into an elective surgical hub. In England, hospitals are run by NHS hospital trusts, with a trust typically running between one and four secondary care hospitals. A surgical team can operate across all hospitals in a trust. Data on where a procedure was conducted in the trust, and whether it was conducted within a hub, are not always recorded accurately. Thus, hubs were defined, and data linked, at a trust rather than a hospital level.

The number of hub trusts increased throughout the study period as new elective surgical hubs were opened. The date the hub started conducting orthopaedic surgery was available and our analysis adjusted for the increasing number of procedures conducted in elective surgical hubs.

### Outcomes

The following outcomes were considered:

• number of HVLC procedures conducted by trainees;
• proportion of all HVLC procedures recorded in the HES database that involved a trainee. This was calculated by dividing the number of procedures involving a trainee (from eLogbook) by the number of procedures recorded in HES for a given variable of interest.

### Variables

The following outcomes were considered:

• grade of surgeon at time of procedure (ST3-8). These data were categorised as ST3-5 (junior trainee) and ST6-8 (senior trainee) for analysis;
• procedure performed (ACLR, BS, TSA, THR, TKR and UKR);
• year of procedure;
• trainee role in the procedure (S-TS, S-TU, P).

### Data management and statistical analyses

Data were analysed using standard statistical software: Microsoft Excel (Microsoft Corp, Redmond, WA, USA), Stata (Stata Corp LLC, College Station, TX, USA) and Alteryx (Alteryx Inc, Irvine, CA, USA). The data presented are largely descriptive, with the statistics used appropriate to the level of the data (interval/ratio, ordinal, categorical).

For data in ISCP, the start date, but not the end date, of each training grade was given. Of 125,579 procedures where trainees conducted the procedure as first surgeon, a procedure was performed after the start of one training grade with no subsequent dates entered for the next sequential training grade in 10,632 (8.5%) cases. In these cases, where the procedure was conducted within 18 months of the last listed start date, the procedure was assumed to have been conducted at that training grade. Likewise, dates of completing ST8 had to be estimated. Trainees starting ST8 before 1 April 2019 were assumed to have completed ST8 (and no longer be a trainee) one year from starting ST8. Trainees starting ST8 on or after 1 April 2019 (and so completing during or after the COVID-19 pandemic) were assumed to have completed ST8 18 months from starting ST8. Procedures conducted after this estimated training end date were assumed not to be conducted by a trainee and excluded.

Changes from 2017–2018 to 2022–2023 in the proportion of procedures involving a trainee were calculated for hub and non-hub trusts. Statistical significance for differences in proportions was evaluated using 95% confidence intervals (CIs), with a 95% CI not containing the value zero indicating significance.

## Results

The data extraction process is summarised in Supplementary material Figure S1 and yielded a dataset for 1,802 ST3-8 trainee surgeons who had conducted at least one of the six included HVLC procedures in a NHS hospital trust in England during the study period. Forty-seven trainee surgeons had only assisted, leaving 1,755 who acted as S-TS, S-TU or P at least once during the study period. These individuals were involved in 245,284 procedures recorded in eLogbook. Details of the procedures undertaken, the role of the trainee and whether the procedure was conducted in a hub trust or a non-hub trust are presented in Table 1. The overwhelming majority of procedures involved the trainee assisting or in a S-TS role (*n*=234,274, 95.5%) and this was consistent across hub (95.2%) and non-hub (95.7%) trusts. TKR and THR were the most common procedure conducted (*n*=160,470, 65.4%); again, this was consistent across hub (64.5%) and non-hub (66.0%) trusts.

**Table 1:** Number of procedures involving trainees that were conducted at trusts hosting or feeding into an elective surgical hub and those not associated with an elective surgical hub

	P*	S-TU*	S-TS*	Assisted
Hub trusts				
ACLR	29 (1.2%)	34 (1.5%)	1,205 (2.7%)	4,475 (9.4%)
BS	371 (15.1%)	354 (16.0%)	6,215 (13.8%)	2,924 (6.2%)
TSA	333 (13.6%)	450 (20.4%)	7,317 (16.3%)	6,459 (13.6%)
THR	776 (31.7%)	460 (20.8%)	14,051 (31.2%)	17,453 (36.7%)
TKR	896 (36.6%)	884 (40.0%)	15,136 (33.6%)	13,040 (27.4%)
UKR	46 (1.9%)	28 (1.3%)	1,102 (2.4%)	3,154 (6.6%)
Total	2,451	2,210	45,026	47,505
Non-hub trusts				
ACLR	85 (2.6%)	59 (1.9%)	1,951 (2.8%)	7,614 (10.6%)
BS	428 (13.2%)	434 (13.9%)	9,731 (14.0%)	4,652 (6.5%)
TSA	431 (13.3%)	950 (30.5%)	9,646 (13.8%)	9,643 (13.4%)
THR	975 (30.2%)	674 (21.6%)	22,704 (32.6%)	26,228 (36.4%)
TKR	1,279 (39.6%)	989 (31.7%)	24,648 (35.4%)	20,277 (28.2%)
UKR	33 (1.0%)	12 (0.4%)	1,043 (1.5%)	3,606 (5.0%)
Total	3,231	3,118	69,723	72,020

ACLR = anterior cruciate ligament reconstruction; BS = bunion surgery; P = performed; S-TS = supervised – trainer scrubbed; S-TU = supervised – trainer unscrubbed; THR = primary total hip replacement; TKR = primary total knee replacement TSA = therapeutic shoulder arthroscopy; UKR = unicompartmental knee replacement

*P, S-TU and S-TS mean the trainee was the first surgeon

Of the 245,284 procedures, 119,525 (48.7%) involved the trainee in an assisting role only. Excluding these left 125,759 (51.3%) procedures which were included in subsequent analysis (see Supplementary material Figure S2 for flow diagram by procedure).

Table 2 shows data categorised as hub and non-hub trusts for the number of trainees, trusts, total procedures, and procedures involving a trainee across the six-year study period. The number of trainees and trusts remained relatively stable over the six years. The number of trusts that were part of a hub at the end of a financial year increased from 28 in 2017–2018 to 62 in 2022–2023, meaning that just over 50% of trusts in England were part of a hub by the end of the study period. The total number of procedures conducted in the NHS in 2022–2023 was only 76.8% of the total conducted in 2017–2018 and the absolute number of procedures involving a trainee in 2022–2023 was only 82.3% of the number in 2017–2018 (Table 2).

**Table 2:** Changes over time in number of trainees, trusts, procedures and procedures involving trainees for each year of the study

	2017–2018	2018–2019	2019–2020	2020–2021	2021–2022	2022–2023	Total
Number of trainees*	932	920	928	818	954	980	–
Number of NHS hospital trusts	122	121	121	122	122	123	–
Number of NHS hospital trusts that were part of an elective surgical hub at the end of the financial year	28	29	31	43	60	62	–
Number of procedures conducted in an elective surgical hub trust^†^	45,972	46,513	44,139	23,942	55,683	66,761	283,010
Number of procedures conducted in a trust not linked to an elective surgical hub^†^	111,803	113,415	105,373	33,104	59,295	54,401	477,391
**Total number of procedures conducted**	**157,775**	**159,928**	**149,512**	**57,046**	**114,978**	**121,162**	**760,401**
Number of procedures conducted in an elective surgical hub trust involving a trainee (% of all procedures)	7,703 (16.8)	7,834 (16.8)	6,850 (15.5)	4,369 (18.2)	10,705 (19.2)	12,226 (18.3)	49,687 (17.6)
Number of procedures conducted in a trust not linked to an elective surgical hub involving a trainee (% of all procedures)	17,895 (16.0)	18,220 (16.1)	15,983 (15.2)	5,205 (15.7)	9,938 (16.8)	8,831 (16.2)	76,072 (15.9)
**Total number of procedures involving a trainee (% of all procedures)**	**25,598 (16.2)**	**26,054 (16.3)**	**22,833 (15.3)**	**9,574 (16.8)**	**20,643 (18.0)**	**21,057 (17.4)**	**125,759 (16.5)**

P = performed; S-TS = supervised – trainer scrubbed; S-TU = supervised – trainer unscrubbed

*Trainees are defined as those acting as first surgeon (S-TU, S-TS or P; trainees assisting in a procedure are not included)

^†^Data for all procedures conducted in England meeting the inclusion criteria, taken from the Hospital Episodes Statistics dataset.

From 2017–2018 to 2022–2023 the proportion of procedures conducted by a trainee increased from 16.8% to 18.3% (difference 1.6%, 95% CI for difference 1.1% to 2.0%) in hub trusts and from 16.0% to 16.2% (difference 0.2%, 95% CI for difference −0.1% to 0.6%) in non-hub trusts, suggesting a significant increase in the proportion of training opportunities in hub trusts but no significant increase in non-hub trusts. This proportional increase in training opportunities in hub trusts post-pandemic is not enough to offset the decline in the absolute number of procedures involving a trainee (absolute number of procedures involving a trainee: 2017–2018, 25,598; 2022–2023, 21,057; Table 2).

**Figure 1:**
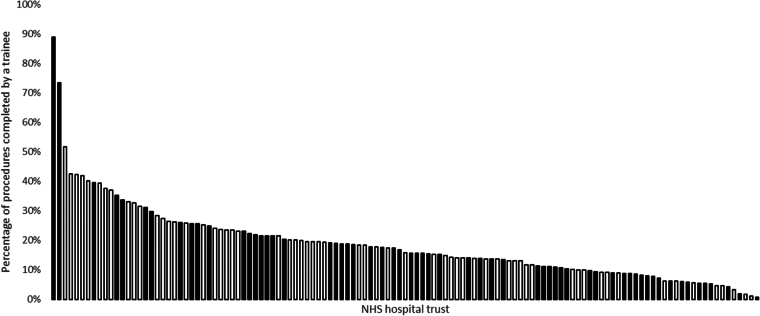
Proportion of all included procedures conducted by trainees in 2022–2023 for each NHS trust in England. Note: Trainees are defined as those acting as firstsurgeon (supervised - trainer scrubbed, supervised - trainer unscrubbed orperformed). Hub trusts are represented by white bars, non-hub trusts arerepresented by black bars. All trusts are anonymised.

Figure 1 shows the variation in the number of all included procedures involving trainees across each trust in England for the financial year 2022–2023. Seven of the top ten trusts for the proportion of procedures conducted by trainees were hub trusts. The proportion of procedures involving trainees varied from 88.9% to 0.7%. Seven trusts and 17 trusts had trainees involved as first surgeon in over 40% and over 30% of all procedures, respectively, regardless of their status as a hub or non-hub trust. The upper quartile value across all 123 trusts in 2022–2023 was 23.6%, with 30 trusts achieving at least this level.

The number of each type of procedure performed and the proportion involving a trainee for each financial year of the study is presented in Table 3. Only for UKR had numbers recovered to above their 2017–2018 level by 2022–2023. Trainee involvement was greatest for BS and lowest for ACLR and UKR. Although all procedures had a higher proportion of procedures involving a trainee in 2022–2023 compared with 2017–2018, the increase was most notable for ACLR and UKR. Trainee involvement in THR and TKR, the highest volume procedures, was only 1–3% higher in 2022–2023 compared with 2017–2018.

**Table 3:** Number of procedures involving trainees for each financial year and for each procedure

Procedure type	Number of procedures	2017–2018	2018–2019	2019–2020	2020–2021	2021–2022	2022–2023	Total
ACLR	Number of procedures conducted	9,142	9,142	8,988	4,363	7,338	8,087	47,060
	Procedures involving a trainee (%)	520 (5.7)	593 (6.5)	493 (5.5)	335 (7.7)	618 (8.4)	804 (9.9)	3,363 (7.1)
BS	Number of procedures conducted	8,920	8,453	7,510	2,355	4,408	4,730	36,376
	Procedures involving a trainee (%)	4,178 (46.8)	3,785 (44.8)	3,529 (47.0)	1,286 (54.6)	2,441 (55.4)	2,314 (48.9)	17,533 (48.2)
TSA	Number of procedures conducted	24,000	20,697	17,501	6,694	11,609	10,682	91,183
	Procedures involving a trainee (%)	4,809 (20.0)	4,071 (19.7)	3,275 (18.7)	1,396 (20.9)	3,132 (27.0)	2,444 (22.9)	19,127 (21.0)
THR	Number of procedures conducted	56,309	60,061	57,035	24,604	48,006	49,578	295,593
	Procedures involving a trainee (%)	7,366 (13.1)	7,915 (13.2)	7,058 (12.4)	3,261 (13.3)	6,855 (14.3)	7,185 (14.5)	39,640 (13.4)
TKR	Number of procedures conducted	54,900	56,659	53,494	17,018	39,281	43,115	264,467
	Procedures involving a trainee (%)	8,466 (15.4)	9,333 (16.5)	8,215 (15.4)	3,027 (17.8)	7,055 (18.0)	7,736 (17.9)	43,832 (16.6)
UKR	Number of procedures conducted	4,504	4,916	4,984	2,012	4,336	4,970	25,722
	Procedures involving a trainee (%)	259 (5.8)	357 (7.3)	263 (5.3)	269 (13.4)	542 (12.5)	574 (11.5)	2,264 (8.8)

ACLR = anterior cruciate ligament reconstruction; BS = bunion surgery; THR = primary total hip replacement; TKR = primary total knee replacement TSA = therapeutic shoulder arthroscopy; UKR = unicompartmental knee replacement

**Figure 2:**
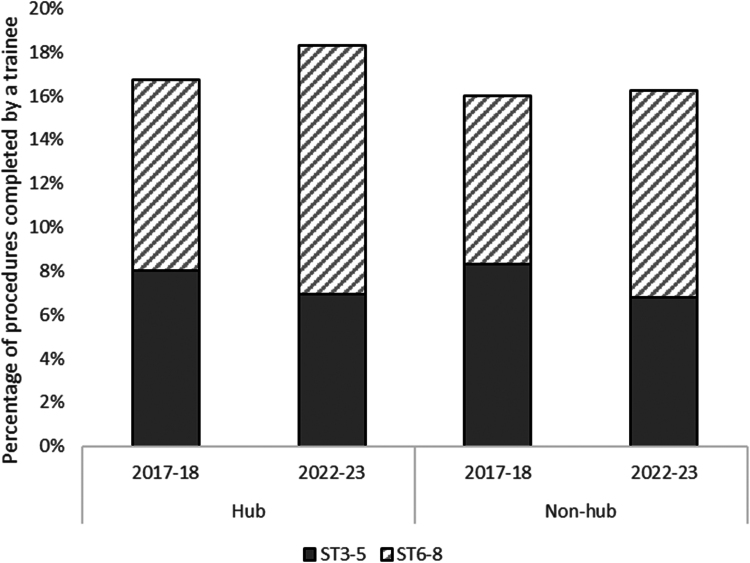
Proportion of procedures conducted by junior (ST3–ST5) and senior (ST6–ST8) orthopaedic trainees in 2017–2018 and 2022–2023 in hub and non-hub sites

Across the study period, trainees in grades ST3-5 conducted 57,067 (45.4%) procedures and trainees in grades ST6-8 conducted 68,692 (54.6%) procedures. Figure 2 shows the variation in training opportunities for each category of training grade in 2017–2018 and 2022–2023. There was a fall from 2017–2018 to 2022–2023 in the proportion of all procedures conducted by ST3-5 trainees in hub (8.0% to 6.9%) and non-hub (8.3% to 6.8%) trusts. However, for ST6-8 trainees, from 2017–2018 to 2022–2023 there was a substantial increase in the proportion of procedures conducted by trainees in hub trusts (8.7% to 11.4%), greater than the increase in non-hub trusts (7.7% to 9.4%).

This increase in the proportion of procedures conducted by ST6-8 trainees in hub trusts was largely responsible for the overall post-pandemic increase in the proportion of procedures conducted by a trainee in hub trusts. The same data, but categorised into each of the six procedures, are presented in Supplementary material Figure S3. A similar pattern to that observed for all procedures combined is seen.

The data for each of the six procedures by year, training grade and hub and non-hub trust are presented in Supplementary material Tables S2 – S7.

## Discussion

Our study provides evidence that, post the COVID-19 pandemic, trusts with access to an elective surgical hub (either hosting or feeding into) have a significantly higher proportion of HVLC orthopaedic procedures where a trainee is involved than they did pre-pandemic and that trusts without access to an elective surgical hub have not seen this increase. However, this proportional increase in training opportunities in hub trusts is not enough to offset the decline in the absolute numbers of training opportunities for orthopaedic registrars from pre-to post-pandemic, due to lower volumes of orthopaedic surgery at NHS hospitals in the post-pandemic period compared with pre-pandemic.

Much of the activity lost to the NHS in the post-pandemic period has been conducted in independent (private) sector hospitals but funded by the NHS with the aim of reducing waiting lists.^12–14^ Furthermore, for some patients, extended waiting lists mean that they have chosen to fund their own surgery privately rather than wait for NHS care.^15^ Since independent sector hospitals in England do not routinely offer training opportunities, care is needed to ensure that sufficient low-complexity procedures remain in NHS hospitals to allow surgeons to be trained.

Of the six procedures we studied, four (BS, TSA, THR and TKR) are classed as indicative procedures that orthopaedic trainees need to conduct to achieve certification of completion of training (CCT). ACLR and UKR are not essential to achieve CCT and are more complex procedures, which may help explain why trainee numbers for these procedures are much lower than for the other four.

The positive impact of hubs on training opportunities are not distributed evenly, with senior trainees benefitting more than junior trainees. There are many factors that could contribute to this, including an unequal distribution of trainees in elective surgical hubs, trainees spending more of their early training years performing trauma cases and attitudes to training among senior surgeons, with junior trainees having fewer opportunities to complete procedures as the first surgeon. Although junior surgeons on average take longer to complete a procedure, there is no definitive evidence that patients operated on by trainees have a higher complication rate.^16,17^

The variation between trusts in the proportion of procedures involving a trainee in our study is clearly unwarranted. Until surgical activity in the NHS increases to at least pre-pandemic levels, the number of surgeons being trained will increase only if more procedures in every trust are viewed as opportunities to involve a trainee.

To achieve GIRFT accreditation, elective surgical hubs must provide training as a central part of their operation. Although hub trusts are outperforming non-hub trusts, most hubs need to do more to support trainees. Since our study end date, the eLogbook has included a question asking whether the procedure was performed at a hub site. This information will be useful to monitor the training provided at hubs and will be a useful metric when site accreditation is being reviewed.

### Strengths and limitations

This is a national study of training opportunities across an entire health service and is the first study of its kind to date. We have been able to demonstrate that elective hubs have had a positive impact on training opportunities for orthopaedic trainees and hope that this information can be utilised by training programme directors when considering placements for their trainees. Our study does have some limitations. Start dates for each training year reflected data recorded by the JCST team but there were some instances when this information was not available. This was felt to be due to a variety of issues including previous training being retrospectively recognised, people who had only part of their training recorded by the JCST and people who left training programmes before completion. Nevertheless, the number of records with missing dates is relatively small, so this should not have substantially biased our findings. We did not have information on the working patterns of the trainees so assumed that all trainees worked full-time, even though this will not be the case for many. As such, we will have underestimated the number of procedures performed by trainees at each grade.

The data in eLogbook are entered by individual trainee surgeons and there will be variation in the accuracy of the information recorded. Double counting of procedures is also possible where more than one trainee was involved in a procedure. Other possible confounders include the number of trainers and trainees at each site, which could impact on the training opportunities available, and the possibility that Annual Review of Competency Progression (ARCP) outcomes may have impacted on the progression of trainees during the COVID-19 pandemic.

## Conclusions

Elective surgical hubs are helping to increase orthopaedic surgical training opportunities, but more needs to be done to ensure surgeons are being trained in sufficient numbers to meet future demand. In the short term, all trusts, but especially those accessing elective surgical hubs, need to increase the number of training opportunities per procedure conducted. In the medium term, procedures lost to the independent sector need to return to the NHS to enable trainees to develop their practice. The hashtag “#NoTrainingTodayNoSurgeonsTomorrow” was utilised during the COVID-19 pandemic to highlight the importance of surgical training.^18^ This message is still relevant.

## Data Availability

This report does not contain patient identifiable data. Data in this report from eLogbook were anonymised at source and cannot be made available directly due to the data request agreement from the JCST. The underlying HES data cannot be made available directly by the authors as the data were obtained under licence/data sharing agreement from NHS Digital. HES data are available from NHS Digital upon application.
